# Has interest in smoking cessation declined during the COVID-19 pandemic?

**DOI:** 10.1177/17579139221136720

**Published:** 2023-08-17

**Authors:** JA Cunningham, C Schell, A Godinho

**Affiliations:** National Addiction Centre, Institute of Psychiatry, Psychology and Neuroscience, Kings College London, London, UK; Institute of Mental Health and Policy Research, Centre for Addiction and Mental Health, Toronto, ON, Canada; Department of Psychiatry, University of Toronto, Toronto, ON, Canada; Institute of Mental Health and Policy Research, Centre for Addiction and Mental Health, Toronto, ON, Canada; Dalla Lana School of Public Health, University of Toronto, Toronto, ON, Canada; Institute of Mental Health and Policy Research, Centre for Addiction and Mental Health, Toronto, ON, Canada

*This article focuses on the impact of the COVID-19 pandemic on smoking behaviours and builds upon work published in 2020 which used Google Trends data to investigate if interest in smoking cessation increased during the beginning of the pandemic.*

At the end of 2020, we began recruiting for a study which examined the effect of mailing a five week supply of nicotine patches free-of-charge to adult smokers in rural communities across Canada, in order to promote smoking cessation.^1^ Based on previous recruitment efforts, we expected to recruit 1252 participants in eight months. Although we recruited the desired sample, it took nearly twice as long as expected. While there are likely a number of contributing factors, we were particularly interested in understanding the impact the COVID-19 pandemic may have had on the motivation to quit smoking and if it has contributed to a decreased interest in smoking cessation.

On 11 March 2020, the World Health Organization (WHO) declared a global pandemic caused by the spread of the severe acute respiratory syndrome coronavirus 2 (SARS CoV-2) and the resultant coronavirus disease 2019 (COVID-19).^2,3^ Health professionals and experts were quick to express concerns related to the risk the respiratory virus posed to people who smoke. Past research involving other respiratory infections (e.g. influenza, MERSCoV) has demonstrated that smokers are more vulnerable to infectious disease^4,5^ and subsequent research specifically addressing the effects of COVID-19 have largely supported this. People who smoke appear to be at greater risk of infection from COVID-19 and if infected are at greater risk of experiencing severe complications including hospitalisation, needing mechanical ventilation, intensive care, and/or having the infection result in death.^5–9^

**Figure:**
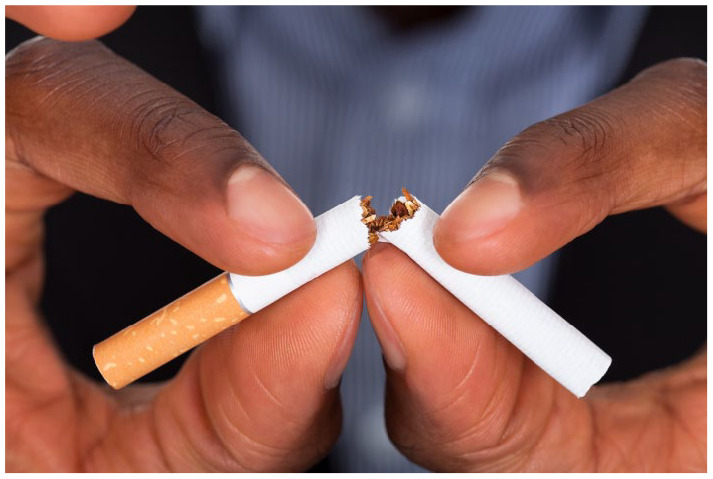


This greater risk has led many smokers to express renewed interest in smoking cessation^8^ and a number of health agencies and experts have recommended using the pandemic as an impetus to encourage smokers to make attempts to quit,^8,10^ as it is an established theory of behaviour change that an increased perceived risk can increase an individual’s motivation to change a behaviour.^8,11^ During the pandemic, there have been targeted education campaigns; however, the actual effectiveness of the messaging remains unclear. One study found that a combined message that explained the role of smoking in increasing COVID-19 severity was most effective at increasing intentions to quit in the next month and in reducing smoking in the next six months, compared to those where the message only focused on the risks associated with COVID-19 exposure.^8^ Conversely, another study found that messages explaining the harms of smoking with and without COVID-19 risks were equally more effective than a control message. These authors note that a single message is unlikely to change an intention or behaviour. Furthermore, they speculate about the role of an individuals’ fatigue in hearing smoking and COVID-19 messaging on their intention to make a quit attempt.^10^

While the effectiveness of COVID-19 messaging may be unclear, an intention to quit smoking is an important step in changing smoking behaviour. Independent of message effectiveness, several studies have reported increased intentions among people who smoke. An MTurk survey of people who use both tobacco cigarettes and e-cigarettes found more than a third of participants reported greater motivation to quit smoking.^12^ Likewise, a representative survey of US adults found 26% of tobacco cigarette and 41% or e-cigarette smokers reported trying to quit.^11^ However, there is also some evidence that smoking has increased, or at least stayed the same among smokers. The same survey of US adults also found 33% of tobacco cigarette and 23% of e-cigarettes users reported increasing their use^11^ and this finding was echoed in the MTurk study where nearly half of respondents reported no change in their use, 15% reported less motivation to quit and 30% increased their use.^12^ Substance use and smoking are well-documented coping mechanisms in response to negative affect,^12^ stressful life events,^11^ and large-scale hardship.^9^ Increased stress related to disruptions caused by the pandemic^7^ and COVID-19-related anxiety^9^ may help explain why some smokers have increased or maintained their smoking behaviour.

It is unclear from these self-reported surveys if smoking cessation is increasing, decreasing, or remaining the same among smokers in light of the COVID-19 pandemic. Google Trends data overcome some of the limitations of self-reports and have been used to estimate interest in smoking cessation during the pandemic. Google Trends allows public access to aggregated data from the Google search engine. The site produces a search volume index graph for each search term and represents interest over time relative to the highest point on the chart (i.e. 100 = peak popularity, 50 = half as popular, 0 = insufficient data).^13^ Google Trends data have been useful in research. For example, researchers used changes in the popularity of search terms related to flu symptoms to identify outbreaks 7–10 days before traditional Centers for Disease Control Surveillance programmes.^14^

**Figure 1:**
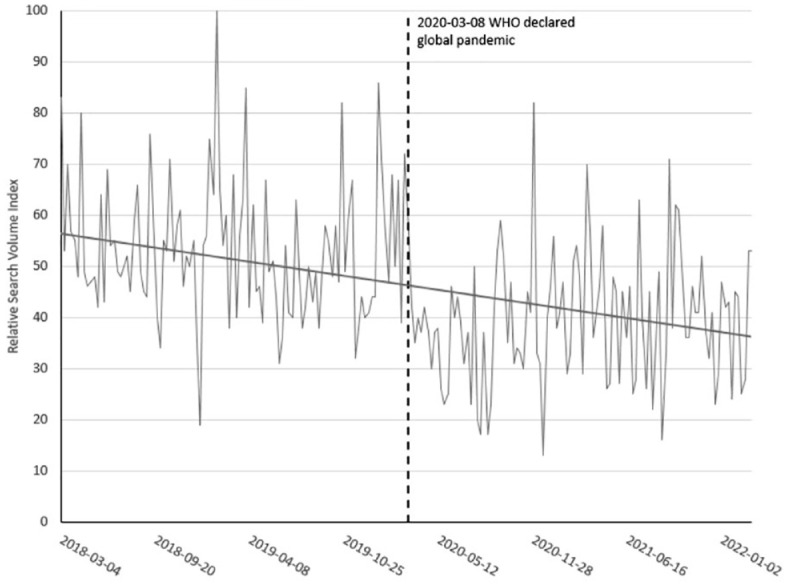
Google Trends relative search volume index for smoking cessation in Canada before and during the COVID-19 pandemic.

Using Google Trends for near real-time monitoring of healthcare seeking behaviour in research is in its early stages and there are some limitations; however, Heerfordt and Heerfordt^4^ used Google Trends to investigate if interest in smoking cessation increased during the beginning of the pandemic (16 January–13 April 2020). While there were reports of more smokers indicating intentions to quit smoking, Google searches for ‘smoking cessation’, ‘quit smoking’, and ‘help quitting’ appeared unchanged during this time.^4^ We extended this analysis and used Google Trends data to compare the relative search index during the two years of the pandemic (March 2020 to March 2022) with the preceding two years (March 2018 to March 2020). We found a significant decrease in the relative search volume index during the pandemic among searches for ‘smoking cessation’ in Canada (mean prepandemic = 52.5, SD = 13.2; mean during pandemic = 38.8, SD = 12.1; *t*(208) = 7.8, *p* < .001; Figure 1).

The approximation methods used by Google Trends to generate the relative search volume index ensure anonymity and user privacy, but is also a limitation as the method has not been clearly shared by the company and may contain inaccuracies or data sampling issues.^3^ Furthermore, search terms are not standardised, and while Google is the most visited website on the Internet,^15^ it is not the only way smokers seek help to quit smoking (e.g. doctors, public health offices, telephone help lines). With these limitations in mind, Google Trends, combined with self-reported survey data reporting increased tobacco use, and our challenges recruiting for a smoking cessation study are concerning. Overall, the impact of COVID-19 and the resulting disruptions caused by the pandemic appear to have had a mixed effect on smoking behaviour. However, if the decrease in searches actually correlates with decreased attempts to quit smoking, clinicians, public health agencies, and researchers will need to monitor this trend and the motivation to quit among smokers in order to ensure that declines in smoking cessation do not become part of our ‘new normal’.
